# Methods for identifying lipoxygenase producing microorganisms on agar plates

**DOI:** 10.1186/2191-0855-2-17

**Published:** 2012-03-26

**Authors:** Antti Nyyssölä, Ruud Heshof, Thomas Haarmann, Jasmin Eidner, Ann Westerholm-Parvinen, Kim Langfelder, Kristiina Kruus, Leo de Graaff, Johanna Buchert

**Affiliations:** 1 VTT Biotechnology, P.O. Box 1500, FIN-02044 VTT, Finland; 2 Wageningen University, Laboratory of Systems and Synthetic Biology, Fungal Systems Biology, Dreijenplein 10, 6703 HB Wageningen, The Netherlands; 3 AB Enzymes GmbH, Feldbergstrasse 78, D-64293 Darmstadt, Germany

## Abstract

Plate assays for lipoxygenase producing microorganisms on agar plates have been developed. Both potassium iodide-starch and indamine dye formation methods were effective for detecting soybean lipoxygenase activity on agar plates. A positive result was also achieved using the β-carotene bleaching method, but the sensitivity of this method was lower than the other two methods. The potassium iodide-starch and indamine dye formation methods were also applied for detecting lipoxygenase production by *Trichoderma reesei *and *Pichia pastoris *transformants expressing the lipoxygenase gene of the fungus *Gaeumannomyces graminis*. In both cases lipoxygenase production in the transformants could be identified. For detection of the *G. graminis *lipoxygenase produced by *Aspergillus nidulans *the potassium iodide-starch method was successful. When *Escherichia coli *was grown on agar and soybean lipoxygenase was applied on the culture lipoxygenase activity could clearly be detected by the indamine dye formation method. This suggests that the method has potential for screening of metagenomic libraries in *E. coli *for lipoxygenase activity.

## Introduction

Lipoxygenases (EC 1.13.11.12, EC 1.13.11.34 and EC 1.13.11.33) are non-heme, iron or manganese containing enzymes which catalyze the oxidation of unsaturated fatty acids containing a 1-*cis*,4-*cis*-pentadiene structure to fatty acid hydroperoxides. Linoleic acid, linolenic acid and arachidonic acid are the natural substrates of lipoxygenases (Su and Oliw 1998; Goldsmith et al. 2002).

Lipoxygenase catalyzed oxidative coupling reactions are utilized extensively in bread making. The addition of lipoxygenase-rich soybean flour results in the production of fatty acid hydroperoxides, which bleach the pigments of wheat flours. Lipoxygenase action also improves the mixing tolerance and rheological properties of the dough. (Frazier et al. 1973; Faubion and Hoseney 1981; Boussard et al. 2012. Furthermore, lipoxygenases have been used for bleaching dairy products such as cheese, milk, butter oil, cream, and whey (de Roos et al., 2006). Alcohols and aldehydes have been produced industrially from the fatty acid hydroperoxides that are formed in the lipoxygenase-catalyzed reactions. These compounds are used for flavouring foods (Whitehead et al. 1995).

Lipoxygenases have been detected in plants Feussner and Wasternack (2002); Baysal and Demirdöven 2007), mammals (Hammarberg et al. 1993), and in eukaryotic (Su and Oliw 1998) and prokaryotic microorganisms (Koeduka et al. 2007; Vance et al. 2004). However, the recombinant production of secreted, stable lipoxygenases at high levels has only been achieved by the expression of the lipoxygenase gene of the fungus *Gaeumannomyces graminis *in *Pichia pastoris *(Cristea et al. 2005).

Many different assays for lipoxygenases have been developed (Axelrod et al. 1981; Williams et al. 1986; Suda et al. 1995; Villafuerte Romero and Barrett 1997; Anthon and Barrett 2001). The majority of these assays are based on detecting the fatty acid hydroperoxides formed, either indirectly by colorimetry or directly by UV-spectroscopy. However, for screening micoorganisms for industrially interesting enzymatic activities, plate assays are in most cases the method of choice, since this allows high-thoughput screening for the colonies expressing active enzyme. To our knowledge no agar plate assays have been developed for screening microorganisms for the production of secreted lipoxygenases. In this paper we present methods for the detection of lipoxygenase production in microorganisms. The test organisms include *Trichoderma reesei, P. pastoris *and *Aspergillus nidulans *expressing the *G. graminis *lipoxygenase gene, as well as the native lipoxygenase producer *G. graminis triciti*.

## Materials and methods

### Cloning the *G. graminis *lipoxygenase gene

The gene encoding the *G. graminis *lipoxygenase AAK81882.1 was codon-optimized and synthesized by GenScript custom gene synthesis service (GenScript Corporation, Piscataway, USA) for expression in *P. pastoris*. To facilitate cloning, *Eco*RI and *Kpn*I restriction sites were added at the 5' and 3' ends of the fragment, respectively. The DNA fragment of the gene synthesized was ligated into the *Eco*RI and *Kpn*I sites of the vector pPICZαA (Invitrogen) bringing the expression under the control of the methanol inducible AOX1 promoter. The native signal sequences were replaced with the prepro sequence of the *Saccharomyces cerevisiae *α-factor secretion signal for secretion of the recombinant protein. *P. pastoris *wild-type strain X-33 (Invitrogen) was transformed by electroporation with the lipoxygenase expression vector that had been linearised with *Pme*I to target the integration into the AOX1 locus. The transformants were plated on YPDS plates containing 100 μl ml^-1 ^Zeocin (Invitrogen) and cultivated at 30 C. Single crossover recombination at the AOX1 locus was verified by PCR using 5' and 3' AOX1 primers.

For expression in *Trichoderma *the *G. graminis *lipoxygenase encoding gene AAK81882.1 was codon-optimized and synthesized by GeneArt (Regensburg, Germany). To facilitate cloning, *Sac*II and *Bam*HI restriction sites were added at the 5' and 3' ends of the fragment, respectively. The DNA fragment of the synthesized gene was ligated into the *Sac*II and *Bam*HI sites of the expression vector pAB100 containing the strong *cbhI*-promoter of *T. reesei*. The native signal sequence of the recombinant lipoxygenase was used for secretion. *T. reesei *strain RH32578 was transformed as described (Penttilä et al. 1987) and transformants were screened for growth on plates with acetamide as sole nitrogen source. Resulting transformants were analyzed by PCR for integration of the lipoxygenase gene into the genome.

The gene encoding the *G. graminis *lipoxygenase AAK81882.1 was codon-optimized for expression in *Aspergillus niger *and synthesized by DNA 2.0 (Menlo Park, USA). For expression the promoter and secretion signal of the *xlnD *gene, from *Aspergillus niger *[GI 74626559] (Van Peij et al. 1997), replaced the native secretion signal of *G. graminis*. With help of the *Xba*I and *Bam*HI restriction sites the synthesized gene was incorperated into a pUC19 vector and was used to transform the *pyrA *strain *A. nidulans *WG505. The transformants were plated on MMS plates (Kusters et al. 1991) for 4 days at 37 C. Positive transformants were analyzed by PCR for verification of integration of the lipoxygenase gene into the genome.

### Lipoxygenase plate assay methods

A 25 mM linoleic acid solution containing 14 mg ml^-1 ^Tween 20 was used in the β-carotene bleaching assay and indamine dye formation assay was prepared as described previously (Anthon and Barrett 2001). All incubations were carried out in the dark at room temperature.

#### β-carotene bleaching

The half-saturated solution of β-carotene in acetone used in the assay was prepared as follows. Five mg of β-carotene was dissolved in 5 ml of acetone. The suspension was mixed thoroughly and centrifuged for 10 min at 25 000 g. The supernatant was diluted with the same volume of acetone to give 50% saturation with respect to β-carotene.

The linoleic acid agarose solution contained 2.3 mM linoleic acid, 1% (w v^-1^) low melting point agarose (Seaplaque) and 50 mM potassium phosphate buffer (pH 7.0). The linoleic acid agarose solution was mixed at a ratio of 39:5 (w v^-1^) with half-saturated solution of β-carotene in acetone and applied as an overlay. This method is based on a previously described lipoxygenase assay of liquid samples (Villafuerte Romero and Barrett 1997).

#### Indamine dye formation

The indamine dye formation assay is based on the chemical reaction described by Ngo and Lenhoff (1980) A 10 mM DMAB [3-(dimethylamine) benzoic acid] stock solution buffered with 200 mM disodium phosphate pH 6.0; the 10 mM MBTH (3-Methyl-2-benzothiazolinone) stock solution and the 0.1 mg ml^-1 ^hematin stock solution buffered with 5 mM NaOH were prepared as described previously (Anthon and Barrett, 2001). An overlay of agarose with linoleic acid and the coloring reagents contained 2.3 mM linoleic acid, 1% (w v^-1^) agarose, 50 mM K-phosphate buffer (pH 7.0), 4.6 mM DMAB, 0.1 mM MBTH, and 1 μg l^-1 ^hematin.

#### Potassium iodide (KI) - starch method

The potassium iodide assay described by Williams et al. (1986) was modified for detecting lipoxygenase activity of agar cultures. A linoleic acid solution was prepared by mixing linoleic acid (700 μl) and Tween 20 (700 μl) with 5 ml water. The resulting emulsion was clarified by adding 1 ml 1 M NaOH and diluted to a final volume of 25 ml. For detecting lipoxygenase activity 100 μl of the linoleic acid solution was added into wells on agar containing 40 g l^-1 ^starch. After incubation, 100 μl of saturated potassium iodide solution and 75 μl of 75% (v v^-1^) acetic acid were added into the wells and mixed.

### Comparison of the sensitivities of the assays

An ammonium sulfate suspension of type V soybean lipoxygenase (sLOX-1) (Sigma) was diluted with 50 mM potassium phosphate buffer at pH 7.0. 25 μl of the lipoxygenase solution was applied into wells in LB-agar in amounts ranging from 8 ng to1 μg lipoxygenase per well. For the the KI-starch method starch was added to the LB-agar as described above. For comparison of the sensitivities, the plates were incubated at room temperature for 2 h and examined visually.

### Cultivation of the microorganisms on agar plates for detection of lipoxygenase activity

The *P. pastoris *transformants were grown at 30°C for 3 days on BMMY (Buffered Methanol Complex Medium, Invitrogen) agar before analysis. The inducer methanol was added daily to the lid of the inverted plate to compensate for loss due to evaporation. The *T. reesei *transformants were grown on potato dextrose agar at 28-30 C. For analysis using the KI-starch method the plates were supplemented with starch as described above. The *A. nidulans *transformants were grown on 50 mM D-xylose minimal medium plates (Pontecorvo et al. 1953) containing 1.2% agar while potato dextrose was omitted to prevent carbon catabolite repression. *G. graminis *var. *tritici *CBS 905.73 was grown on potato dextrose agar at 25°C. For analysis by the KI-starch method the medium was supplemented with starch as described above. For the indamine dye formation assay the coloring reagents were applied as an agarose overlay.

### Effect of the linoleic acid oxidation products on the growth of *E. coli*

To study the inhibitory effects of the reaction products of lipoxygenase catalyzed oxidation, linoleic acid was dispersed by mechanical homogenization into LB-agar at a concentration of 0.4% (w v^-1^). 4 μl of a solution containing 0.5, 1.8 and 7.2 μg of soybean lipoxygenase (Sigma L-7395) in 50% (w v^-1^) glycerol, 100 mM Na-phosphate buffer, pH 7.0 was applied into wells present in the agar and the plates were incubated for 30 min at room temperature. *E. coli *(XL1-blue) suspensions were spread on the plates after the incubation and the cells were grown overnight.

### Detection of lipoxygenase activity by the indamine dye formation method in the presence of *E. coli *cells

Wells were punched into LB-agar plates and were inoculated with 400 μl of *E. coli *(XL1-blue) suspension in LB medium and the cells were cultivated over-night at 37°C as a lawn on the plates sLOX-1 (Sigma) was diluted with 50 mM potassium phosphate buffer, pH 7.0, and 25 μl of the lipoxygenase solution was applied into the wells in the agar at final amounts ranging from 8 ng to1 μg. After the enzyme solutions had absorbed, the indamine dye formation agarose was applied either as a single layer or sequentially as two layers of equal volume. In the latter case the first layer contained 4.6 mM linoleic acid and 100 mM K-phosphate buffer pH 7.0 and the second layer contained 9.2 mM DMAB, 0.2 mM MBTH and 2 μg hematin. After the application of the first layer the plates were incubated overnight at room temperature and the second layer was applied. Experiments both with and without 0.5 mM EDTA in the overlays were carried out.

## Results

### Comparison of the assays for detection of soybean lipoxygenase

Lipoxygenase catalyzed formation of hydroperoxides resulted in a violet-blue color with the indamine dye formation method, a brown color with the potassium iodine-starch method and bleached halos in the yellow-orange agarose background with the β-carotene bleaching method.

**Figure 1:**
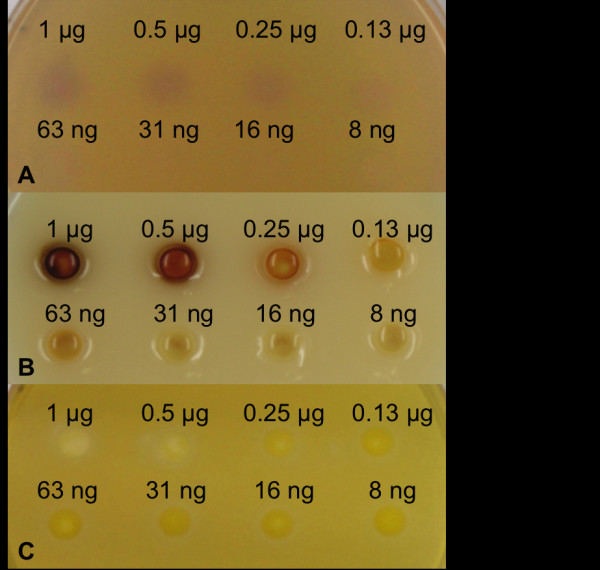
**Comparison of the sensitivities of (A) indamine dye formation, (B) KI-starch, and (C) β-carotene bleaching methods**. Different concentrations of sLOX-1 were applied on the agar plates. The plates were incubated for 2 h at room temperature.

To compare the three methods soybean lipoxygenase was applied on the agar plate and analyzed. As shown in Figure 1, the soybean lipoxygenase activity on linoleic acid was detectable by all three colorimetric methods. The sensitivities of the indamine dye formation and KI - starch methods were of the same order, the detection limits being between 63 and 130 ng soybean lipoxygenase. The β-carotene bleaching method appeared to be the least sensitive of the three methods tested, since the detection limit was between 0.25 μg and 0.5 μg of soybean lipoxygenase. Therefore, β-carotene bleaching was not used for the assays for lipoxygenase activity in further experiments.

### Detection of lipoxygenase production by microbial transformants

**Figure 2:**
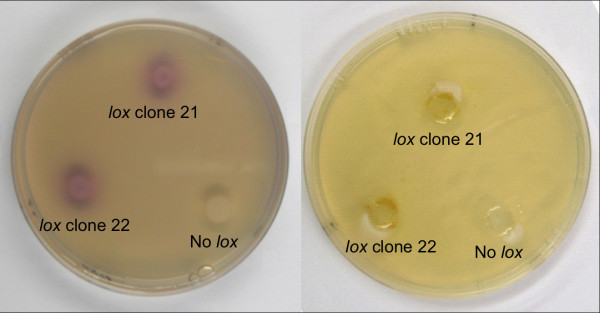
**Lipoxygenase detection of *P. pastoris *transformants expressing the lipoxygenase of *G. graminis *(A) analyzed by the indamine dye formation method and (B) analyzed by the KI-starch method**.

*P. pastoris, T. reesei *and *A. nidulans *transformants with and without the lipoxygenase gene of *G. graminis *were grown on agar plates and were induced as described in Materials and Methods. The transformants were analyzed for lipoxygenase activity using the KI-starch and indamine color formation methods. As shown in Figure 2, both *P. pastoris *transformants with the lipoxygenase gene gave clear positive signals with both methods. For the assay using the KI-starch method it proved to be necessary to grow the *Pichia *transformants on plates composed of a layer without starch on top and a layer with it on the bottom. Wells were punched in the upper layer in the vicinity of the colonies and the reagents added. Possibly the starch used contained traces of free sugars that caused carbon catabolite repression of expression.

**Figure 3:**
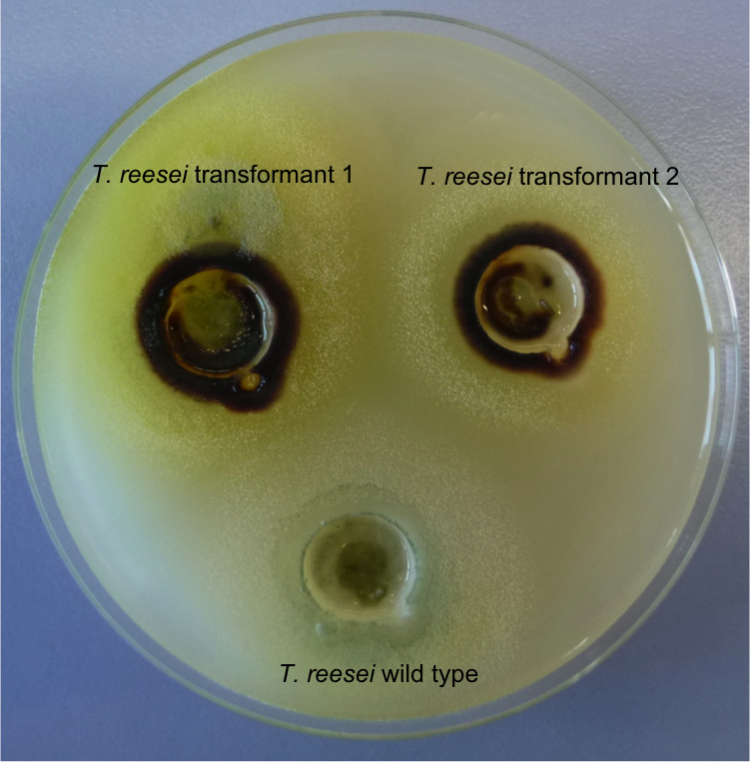
**Detection of *G. graminis *lipoxygenase activity on *T. reesei *transformants grown on plate**. Positive transformants show a brown color around the mycelium.

**Figure 4:**
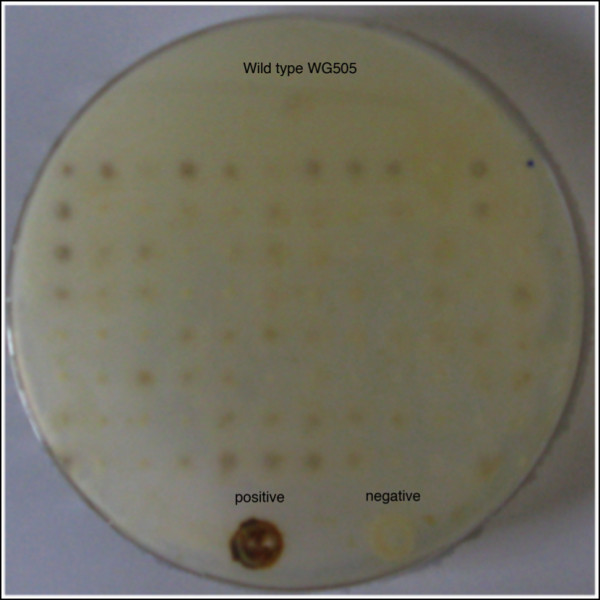
**Detection of *G. graminis *lipoxygenase activity in *A. nidulans *transformants using the KI-starch assay**. Positive transformants are visualized as brown dots in the plate.

Figure 3 shows a clear brown colour around the mycelium of *T. reesei *while this was not found for the wild type showing the plate assay can also be used for this organism. For the plate assays using *A. nidulans *potato dextrose was replaced by D-xylose to prevent inhibition of lipoxygenase by carbon catabolite respression. In these transformants the D-xylose inducible *xlnD *promoter (Van Peij et al. 1997) was used for the expression of the lipoxygenase. The results of the assay are shown in Figure 4, positive transformants for lipoxygenase activity are visible as a brown colony while wild type did not stain. The indamine color assay was not successful for *A. nidulans*, which is probably due to the low expression level of enzyme in this organism.

### Effects of lipoxygenase reaction products on growth of *E. coli *and detection of lipoxygenase activity in the presence of *E. coli *cells

**Figure 5:**
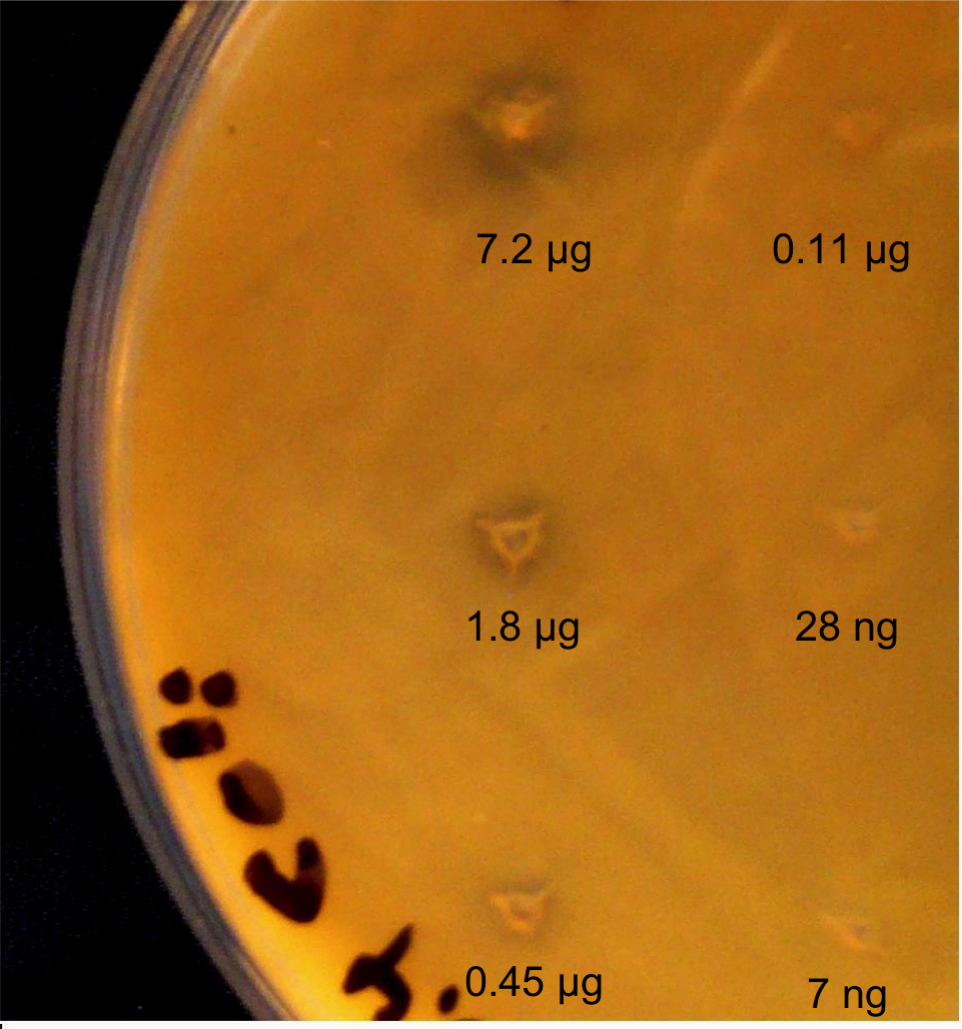
**Overnight growth *E. coli *on linoleic acid agar supplemented with sLOX-1**. The plates were incubated for 30 min at room temperature in the presence of different amounts of the lipoxygenase and inoculated with the cell suspension.

To study the effect of the oxidation products of linoleic acid on the growth of *E. coli*, soybean lipoxygenase was applied on linoleic acid LB-agar. After incubation, *E. coli *cells were grown overnight as a lawn on the plates. As shown in Figure 5 clear growth inhibiting zones could be detected on the plates around the soybean lipoxygenase (between 0.11 μg and 7.2 μg of the enzyme preparate) application points.

**Figure 6:**
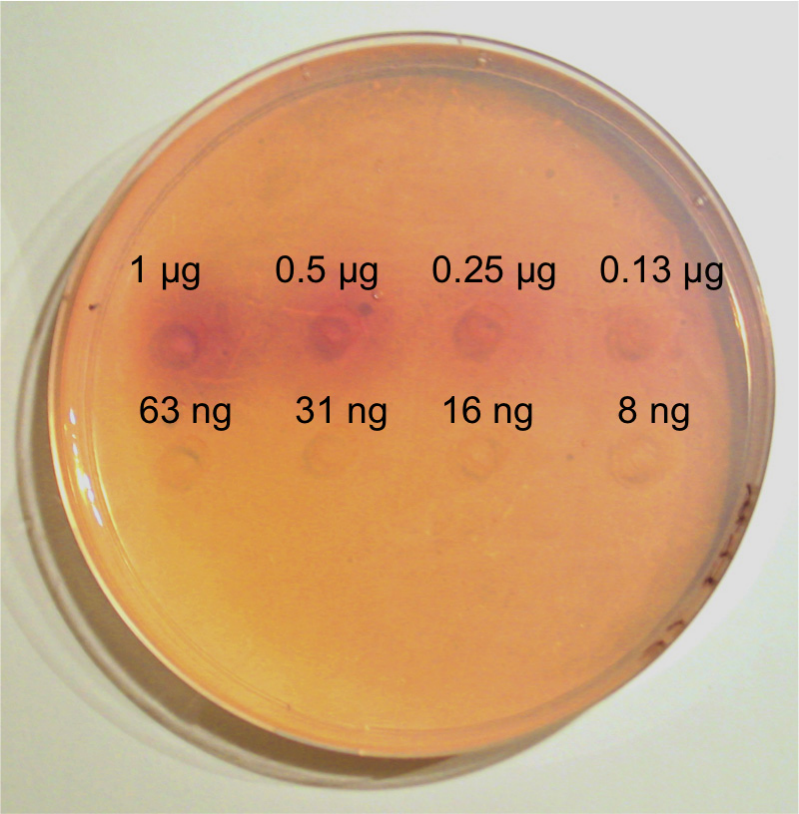
**Detection of sLOX-1 applied on a lawn of *E. coli *cells by the indamine dye formation method**. The substrate and coloring reagent agaroses were supplemented with 0.5 mM EDTA and applied sequentially.

The possibility that *E. coli *cells interfere with the lipoxygenas activity was investigated by applying soybean lipoxygenase on a lawn of growing cells. When all indamine dye formation reagents were added as a single layer only between 0.5 μg and 1 μg soybean lipoxygenase could be detected after 5 h of incubation. When the plates were incubated overnight the blue indamine color had disappeared. However, when the linoleic acid agarose was added first, followed by an overnight incubation and addition of the coloring reagent agarose, the violet-blue color was clearly visible at between 63 ng and 130 ng of soybean lipoxygenase (Figure 6). In both cases the addition of 0.5 M EDTA intensified the signal (data not shown).

## Discussion

The methods used in the current work for identification of lipoxygenase producing transformants on agar plates are all based on the detection of the linoleic acid hydroperoxides formed in the lipoxygenase catalyzed reaction. In the indamine dye formation method 3-methyl-2-benzothiazolinone (MBTH) is coupled oxidatively with 3-(dimethylamino) benzoic acid (DMAB) in a hematin catalyzed reaction, which results in the generation of the indamine dye (Anthon and Barrett 2001). In the potassium iodide starch method the fatty acid hydroperoxides formed oxidize iodide to iodine, which in turn reacts with starch (Williams et al. 1986). β-Carotene can be used for lipoxygenase detection, since it is bleached by the fatty acid hydroperoxides (Villafuerte Romero and Barrett 1997).

The indamine dye formation and KI-starch methods appeared to be more sensitive than the β-carotene bleaching method for detection of lipoxygenase activity. The poor sensitivity of the β-carotene bleaching method has also been established in a previous report in which different liquid assays for detecting lipoxygenase activity from vegetable extracts were compared (Villafuerte Romero and Barrett 1997).

The reaction products of lipoxygenase-catalyzed oxidation of linoleic acid appeared to be toxic for *E. coli *cells. It has also been shown previously that fatty acid hydroperoxides and their degradation products (oxylipins) impair the growth of many microbial plant pathogens (Prost et al., 2005). If linoleic acid is present in the growth medium from the onset of the cultivation, the lipoxygenase producers may thus not be able to survive. Linoleic acid can also be spontaneously degraded *via *auto-oxidation during longer incubations (Seo et al. 1999). For these reasons, we chose to omit linoleic acid from the agar medium. Lipoxygenase positive *T. reesei *and *P. pastoris *transformants expressing the *G. graminis *lipoxygenase gene could be clearly identified by both KI-starch and indamine dye formation methods. These methods could also most likely be used for other microbial production hosts. For the *A. nidulans *transformants only the KI-starch assay was successful. The indamine dye formation method did not show clear results for detection of lipoxygenase (data not shown).

A potential problem in screening *E. coli*-hosted metagenomic libraries is that the cells are able to use the lipoxygenase substrate, linoleic acid, for growth. This problem can, however, most probably be overcome by disrupting genes encoding fatty acid transport and/or β-oxidation present in the *fad *regulon (DiRusso and Nyström, 1998). However, as stated above, adding linoleic acid to the medium may not be feasible, because positive transformants can produce toxic substances *via *the lipoxygenase catalyzed oxidation of the substrate.

Preferably tens of thousands of metagenomic library colonies would have to be screened to find lipoxygenase activity. Furthermore, it is typical for metagenomic expression libraries that the activities are low and may be detectable only after prolonged cultivations (Uchiyama and Miyazaki 2009). The use of the KI-starch method is problematic for the detection of lipoxygenase producing transformants of metagenomic libraries, since the color reaction takes place under very acidic conditions. Iodine is also a well-known microbicide (Vasudevan and Tandon 2010). We chose therefore the indamine dye formation method for investigation whether lipoxygenase activity can be detected in the presence of *E. coli *cells. If linoleic acid and the coloring reagents were applied as one layer on the *E. coli *culture supplemented with soybean lipoxygenase, the violet-blue color was for unknown reasons only transiently visible. However, when the substrate agarose was applied first, followed by incubation and application of the coloring agarose, the soybean lipoxygenase catalyzed reaction was clearly detectable. EDTA intensified the signal, possibly because it chelates metal ions, which can catalyze hydroperoxide degradation.

It can be concluded that the KI-starch and the indamine dye formation method can both be used for detection of lipoxygenase producing transformants of *P. pastoris *and *T. reesei*, and possibly also of other microbial hosts. Furthermore, the indamine dye formation method holds promise for identification of lipoxygenase positive transformants of *E. coli*-hosted metagenomic libraries. However, at the present the detection of lipoxygenase production by natural isolates growing on agar remains a challenge.

## Competing interests

The authors declare that they have no competing interests.
